# Quantifying the Evolutionary Constraints and Potential of Hepatitis C Virus NS5A Protein

**DOI:** 10.1128/mSystems.01111-20

**Published:** 2021-04-13

**Authors:** Lei Dai, Yushen Du, Hangfei Qi, Christian D. Huber, Dongdong Chen, Tian-Hao Zhang, Nicholas C. Wu, Ergang Wang, James O. Lloyd-Smith, Ren Sun

**Affiliations:** a CAS Key Laboratory of Quantitative Engineering Biology, Shenzhen Institute of Synthetic Biology, Shenzhen Institutes of Advanced Technology, Chinese Academy of Sciences, Shenzhen, China; b Cancer Institute, ZJU-UCLA Joint Center for Medical Education and Research, The Second Affiliated Hospital, Zhejiang University School of Medicine, Hangzhou, China; c Department of Molecular and Medical Pharmacology, University of California Los Angeles, Los Angeles, California, USA; d Department of Ecology and Evolutionary Biology, University of California Los Angeles, Los Angeles, California, USA; e Department of Biochemistry, University of Illinois at Urbana-Champaign, Urbana, Illinois, USA; UT Southwestern Medical Center

**Keywords:** DFE, deep mutational scanning, drug resistance, fitness landscape, HCV, viral evolution

## Abstract

RNA viruses, such as hepatitis C virus (HCV), influenza virus, and SARS-CoV-2, are notorious for their ability to evolve rapidly under selection in novel environments. It is known that the high mutation rate of RNA viruses can generate huge genetic diversity to facilitate viral adaptation. However, less attention has been paid to the underlying fitness landscape that represents the selection forces on viral genomes, especially under different selection conditions. Here, we systematically quantified the distribution of fitness effects of about 1,600 single amino acid substitutions in the drug-targeted region of NS5A protein of HCV. We found that the majority of nonsynonymous substitutions incur large fitness costs, suggesting that NS5A protein is highly optimized. The replication fitness of viruses is correlated with the pattern of sequence conservation in nature, and viral evolution is constrained by the need to maintain protein stability. We characterized the adaptive potential of HCV by subjecting the mutant viruses to selection by the antiviral drug daclatasvir at multiple concentrations. Both the relative fitness values and the number of beneficial mutations were found to increase with the increasing concentrations of daclatasvir. The changes in the spectrum of beneficial mutations in NS5A protein can be explained by a pharmacodynamics model describing viral fitness as a function of drug concentration. Overall, our results show that the distribution of fitness effects of mutations is modulated by both the constraints on the biophysical properties of proteins (i.e., selection pressure for protein stability) and the level of environmental stress (i.e., selection pressure for drug resistance).

**IMPORTANCE** Many viruses adapt rapidly to novel selection pressures, such as antiviral drugs. Understanding how pathogens evolve under drug selection is critical for the success of antiviral therapy against human pathogens. By combining deep sequencing with selection experiments in cell culture, we have quantified the distribution of fitness effects of mutations in hepatitis C virus (HCV) NS5A protein. Our results indicate that the majority of single amino acid substitutions in NS5A protein incur large fitness costs. Simulation of protein stability suggests viral evolution is constrained by the need to maintain protein stability. By subjecting the mutant viruses to selection under an antiviral drug, we find that the adaptive potential of viral proteins in a novel environment is modulated by the level of environmental stress, which can be explained by a pharmacodynamics model. Our comprehensive characterization of the fitness landscapes of NS5A can potentially guide the design of effective strategies to limit viral evolution.

## INTRODUCTION

In our evolutionary battles with microbial pathogens, RNA viruses are among the most formidable foes. HIV-1 and hepatitis C virus (HCV) acquire drug resistance in patients under antiviral therapies. Influenza virus, Ebola virus, and SARS-CoV2 cross the species barrier to infect human hosts. Understanding the evolution of RNA viruses is therefore of paramount importance for developing antivirals and vaccines and assessing the risk of future emergence events (1–3). Comprehensive characterization of viral fitness landscapes, and the principles underpinning them, will provide us with a map of evolutionary pathways accessible to viruses and guide our design of effective strategies to limit antiviral resistance, immune escape, and cross-species transmission (4–6).

Although the concept of fitness landscapes has been around for a long time (7), their properties in real biological systems are still under active investigation. Previous empirical studies of fitness landscapes have been constrained by limited sampling of sequence space. In a typical study, mutants are generated by site-directed mutagenesis and assayed for growth rate individually. We and others have recently utilized a high-throughput technique, often referred to as “deep mutational scanning” or “quantitative high-resolution genetics,” to profile the fitness effect of mutations by integrating deep sequencing with selection experiments *in vitro* or *in vivo* (8–14). This application of next-generation sequencing has raised the exciting prospect of large-scale fitness measurements (15–18) and a revolution in our understanding of molecular evolution (19).

The distribution of fitness effects (DFE) of mutations is a fundamental entity in genetics and reveals the local structure of a fitness landscape (12, 20–29). Deleterious mutations are usually abundant and impose severe constraints on the accessibility of fitness landscapes. In contrast, beneficial mutations are rare and provide the raw materials of adaptation. Quantifying the DFE of viruses is crucial for understanding how these pathogens evolve to acquire drug resistance and surmount other evolutionary challenges.

Previously, most empirical studies of the DFE have been performed in a single, static environment (20, 21). A central challenge is to characterize the DFE, and its determinants, in fluctuating or heterogeneous environments where evolution typically occurs (e.g., fluctuating drug concentrations or a gradient across space). More attention has been paid to this area recently. For bacteria, the fitness effects of mutations at different drug concentrations, or under physical and chemical stress, have been studied (30–32). One study has demonstrated that drug concentration modulates the shape of the DFE and determines the evolvability under new environments (33). In another study, the implications of differing drug concentrations on the adaptive landscape have been examined in the context of resistance evolution (34). For viruses, the fitness effects of mutations have been measured across different hosts (35–37). The shape of the DFE of viruses was inferred from experimentally passaged populations (38) and from patient data (39), but not quantified systematically. Combining quantitative high-resolution genetics with different selection conditions will provide a more comprehensive investigation of the DFE under varying levels of positive selection.

In this study, we profile the DFE of ∼1,600 single amino acid substitutions in a drug-targeted viral protein by coupling a selection experiment of a mutant library and deep sequencing. We show that the replication fitness of virus mutants is correlated with the pattern of conservation in patient-derived HCV sequences, suggesting that amino acid sites with high fitness costs are often highly conserved. Combined with simulations of protein stability, we confirm that protein stability is a major determinant of the deleterious effect of mutants and imposes a strong constraint to viral evolution. Furthermore, we examine the changes in DFE under varying levels of environmental stress by tuning the concentration of an antiviral drug. The distribution of beneficial fitness effects of mutations shifts with the increase of environmental stress, in accordance with theoretical predictions (40).

## RESULTS

### Profiling the fitness landscape of the drug-interacting domain of HCV NS5A protein.

The system used in our study is hepatitis C virus (HCV; genotype 2a. J6/JFH1 chimera), a positive-sense single-stranded RNA virus with a genome of ∼9.6 kb. HCV has been studied extensively in the past 2 decades in patients and in the laboratory and provides an excellent model system to study viral evolution. Previously, we constructed a saturation mutagenesis library with all single amino acid substitutions in domain IA (amino acids 18 to 103) of HCV NS5A protein (11). This domain is the target of several directly acting antiviral drugs, including the potent HCV NS5A inhibitor daclatasvir (DCV) (41). Here, we utilized the same plasmid library to further study the DFE of mutations and examine its adaptive potential under various drug selection pressures through a series of new selection experiments. We observed 2,520 nonsynonymous mutations in the plasmid library, as well as 105 synonymous mutations. After transfection to reconstitute mutant viruses, we performed selection in an HCV cell culture system (42, 43). The relative fitness (RF) of a mutant virus was calculated based on the changes in frequency of the mutant virus and the wild-type virus after one round of selection in cell culture (Fig. S1A). In our selection experiment, we grew 5 small sublibraries (∼500 mutants each) separately to reduce the noise in fitness measurements (see Materials and Methods). The fitness data reported in this study are highly correlated with the previously reported independent experiment (Fig. S1B and C) (11).

10.1128/mSystems.01111-20.1 FIG S1 Experimental workflow of high-throughput fitness assays and comparison across experiments. (A) We performed the selection of the mutant virus library using the HCV cell culture system. Viral RNA was extracted 6 days after transfection or 6 days after infection and reverse transcribed into cDNA. The mutated region in NS5A protein was amplified by PCR and sequenced by Illumina HiSeq. The relative fitness of a mutant virus to the wild-type virus was calculated based on the frequency of the mutant virus and the wild-type virus at round 1 (6 days posttransfection) and round 2 (6 days postinfection). See Materials and Methods for more details. (B) The fitness data reported in this study are highly correlated with an independent selection experiment using the same library (11). (C) The fitness values estimated from the changes in mutant frequency between round 1 and round 2 are highly correlated with estimates based on round 0 and round 1. Black lines represent the fits by linear regression. Download FIG S1, TIF file, 1.2 MB. Copyright © 2021 Dai et al. 2021 Dai et al. https://creativecommons.org/licenses/by/4.0/ This content is distributed under the terms of the Creative Commons Attribution 4.0 International license.

**FIG 1:**
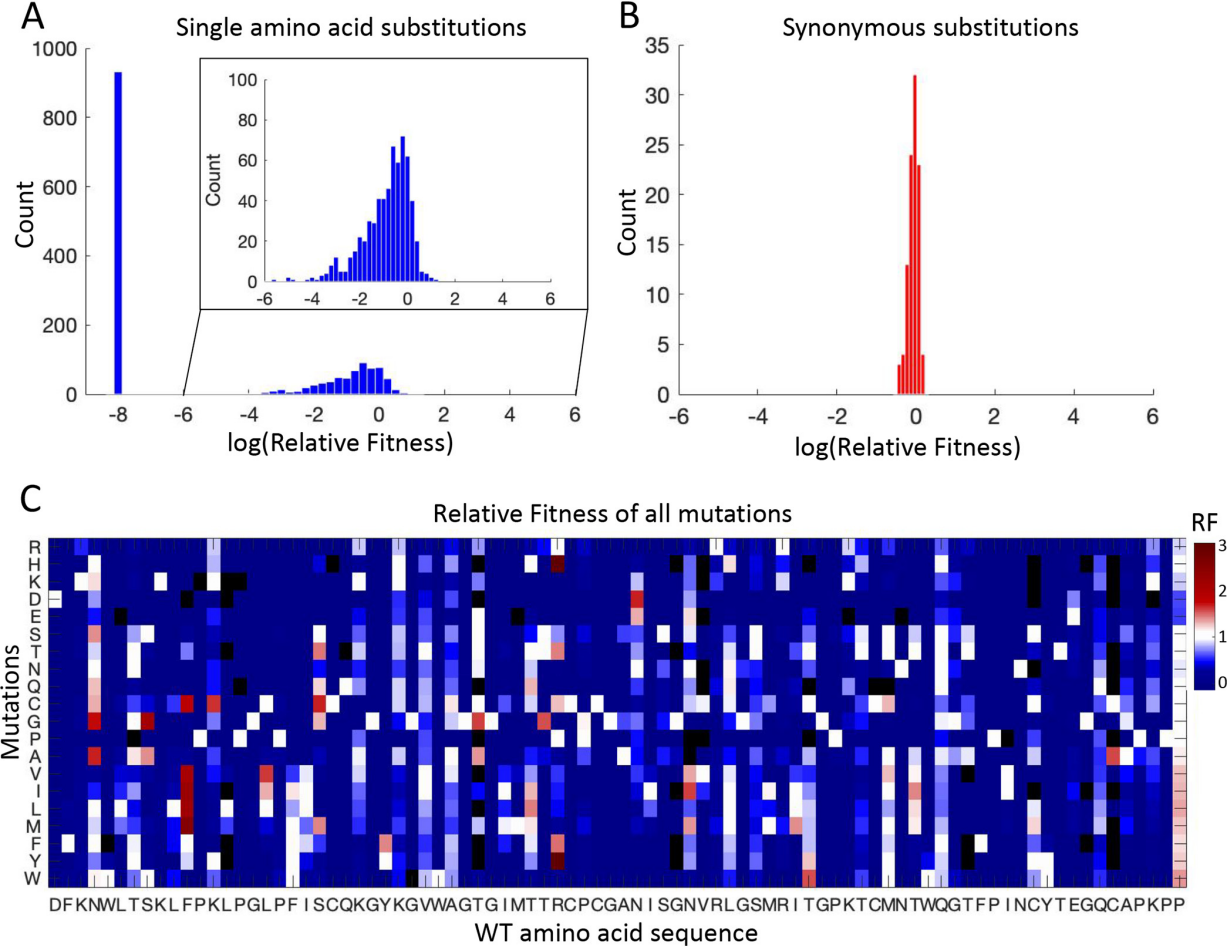
Distribution of fitness effects (DFE) of single amino acid substitutions in domain IA of HCV NS5A protein without drug selection. (A) DFE of single amino acid substitutions. The *x* axis shows the log transformed relative fitness. Lethal mutations are shown at –8. A zoom-in view shows the nonlethal portion of substitutions. (B) DFE of synonymous substitutions, which is centered at 0 for log transformed relative fitness. (C) The Heatmap shows the relative fitness of all mutations. Lethal mutations are shown in dark blue (relative fitness = 0). Mutations that were filtered due to low frequency in the plasmid library (unknown relative fitness) are shown in black.

Our experiment provides a comprehensive profiling of the fitness effect of single amino acid substitutions (1,565 out of 1,634 possible substitutions, after filtering out low-frequency mutants in the plasmid library). We grouped together nonsynonymous mutations leading to the same amino acid substitution (Data set S1). As expected, the fitness effects of synonymous mutations were nearly neutral, while most nonsynonymous mutations were deleterious (Fig. 1A and B). The RF of all mutations is shown with the heatmap in Fig. 1C. We found that the majority of single amino acid mutations had fitness costs, and more than half of them were found to be significantly deleterious, or “lethal” (shown at –8 for Fig. 1A; Materials and Methods). The fraction of lethal mutations is 59.5% (932/1,565) for single amino acid substitutions and 95.1% (77/81) for nonsense mutations with known RF. As NS5A is essential for viral replication, the nonsense mutations should be detrimental. The four nonsense mutations (4/81) that were not identified to be lethal in our profile may due to an experimental artifact that is inevitable in high-throughput genetic screening studies (14, 44). The low tolerance of nonsynonymous mutations in HCV NS5A, which is an essential protein for viral replication, is consistent with previous small-scale mutagenesis studies of RNA viruses (45). Our data support the view that RNA viruses are very sensitive to the effect of deleterious mutations, possibly due to the compactness of their genomes (46, 47).

10.1128/mSystems.01111-20.10 DATA SET S1 Fitness of all single amino acid substitutions fitness_singleaa.txt mutation: amino acid substitutions rf_0: relative fitness, [DCV] = 0 rf_10: relative fitness, [DCV] = 10 pM rf_40: relative fitness, [DCV] = 40 pM rf_100: relative fitness, [DCV] = 100 pM. Download Data Set S1, TXT file, 0.03 MB. Copyright © 2021 Dai et al. 2021 Dai et al. https://creativecommons.org/licenses/by/4.0/ This content is distributed under the terms of the Creative Commons Attribution 4.0 International license.

Using the distribution of fitness effects of synonymous mutations as a benchmark for neutrality, we identified that only 2.4% (37/1,565) of single amino acid mutations are beneficial (Materials and Methods). The estimated fraction of beneficial mutations is consistent with previous small-scale mutagenesis studies of viruses, including bacteriophages, vesicular stomatitis virus, etc. (20, 45, 48, 49). Our results indicate that HCV NS5A protein is under strong purifying selection, suggesting that viral proteins are highly optimized in their natural conditions.

### Deleterious mutations as evolutionary constraints.

**FIG 2:**
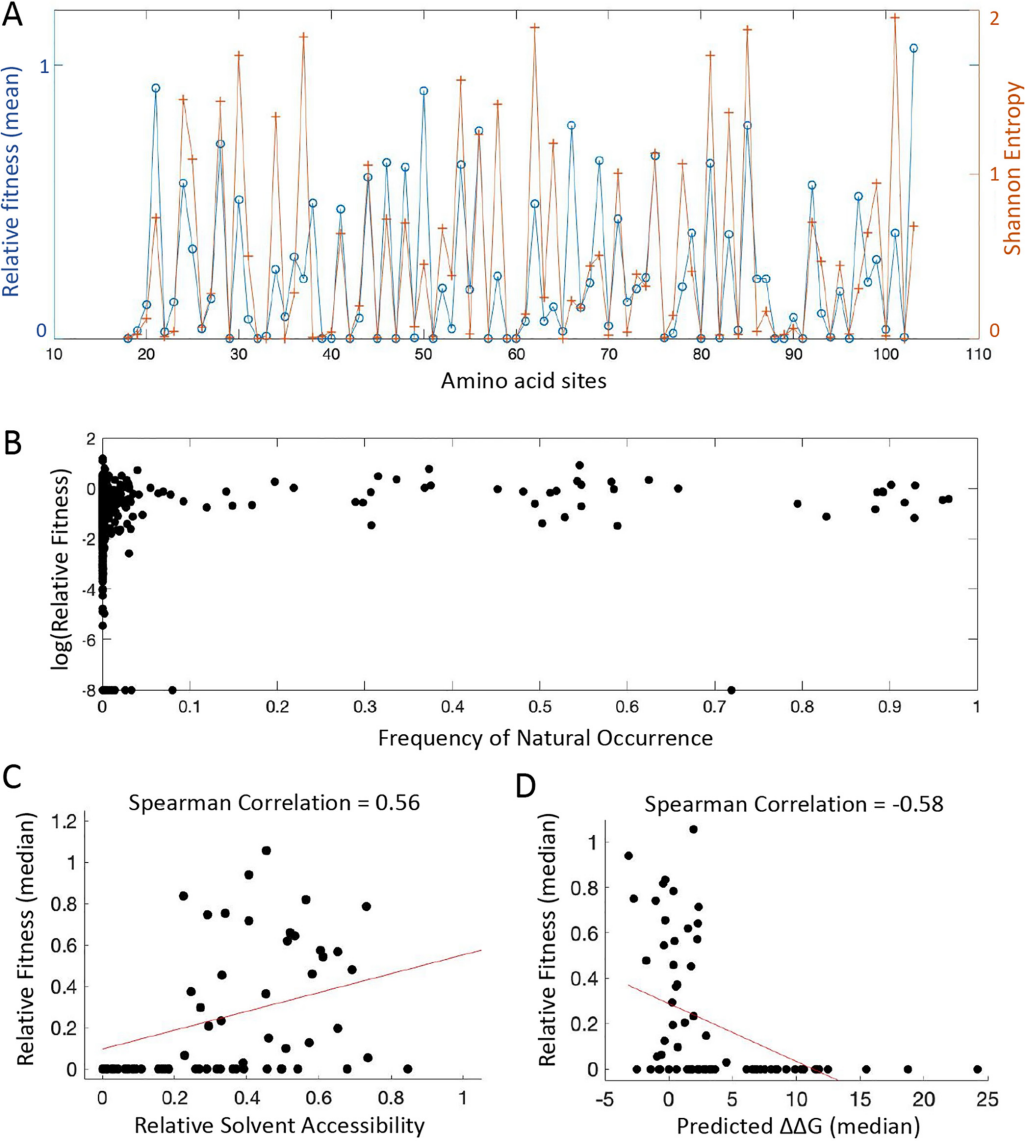
Mutations with deleterious fitness effects reveal constraints of protein evolution. (A) The pattern of sequence conservation observed in patient sequences is highly correlated with the replication fitness measured in cell culture. The blue line shows the average relative fitness for each residue considering all mutations; the orange line shows the Shannon entropy. (B) The Scatterplot shows the frequency of natural occurrence and the log transformed relative fitness for individual mutants. (C) Mutations at amino acid sites with lower solvent accessibility tend to incur larger fitness costs. The relative solvent accessibility for each residue is significantly correlated with median relative fitness (Spearman’s ρ = 0.56, *P* = 3.4 × 10^−7^). (D) Mutations at amino acid sites with larger effects on destabilizing protein stability (predicted ΔΔG > 0) tend to reduce the viral replication fitness. Changes in folding free energy ΔΔG (Rosetta energy unit) of the NS5A monomer were predicted by PyRosetta (PDB: 3FQM). The median predicted ΔΔG at each amino acid site is shown. The median fitness of observed mutants at each amino acid site is shown. In panels C and D, red lines represent the fits by linear regression and are only used to guide the eye.

Mutations that severely reduce replication fitness impose constraints on the evolution of viruses and are less likely to contribute to adaptation through gain of function. We analyzed the sequence diversity of HCV sequences identified in patients from the HCV sequence database of Los Alamos National Lab and the European HCV Database (euHCVdb) (Materials and Methods). To avoid biases toward specific genotypes, we included ∼2,600 sequences from all HCV genotypes in analysis. The sequence diversity at each site was highly correlated with the replication fitness (the mean fitness of observed mutants at each site) measured in our study (Fig. 2A; Spearman’s ρ = 0.81, *P* = 1.75 × 10^−21^). The amino acid sites with high fitness costs were often highly conserved, such as residues 32, 33, 39, 57, 59, 60, 76, 88, 91, 94, et al. We also calculated the frequency of natural occurrence for all mutations and noticed that the majority of mutations with a frequency of >0.1 were relatively neutral in replication fitness (Fig. 2B). Conversely, mutations that do not occur in nature (frequency of 0) may not be lethal for replication fitness, pointing to the potential limited sampling of natural sequences.

To understand the biophysical basis of mutational effects (50), we took advantage of the available structural information. The crystal structure of NS5A domain I is available, excluding the amphipathic helix at the N terminus (51, 52). We calculated the relative solvent accessibility of all residues and found that the fitness effects of deleterious mutations at buried sites (i.e., with lower solvent accessibility) were more pronounced than those at surface-exposed sites (Fig. 2C, Fig. S2A) (53). Residues with average fitness of <0.2 showed a lower relative solvent accessibility (Fig. S2B). Moreover, we performed simulations of protein stability for individual mutants using PyRosetta (Materials and Methods) (54, 55). A mutation with ΔΔG of >0, i.e., shifting the free energy difference to favor the unfolded state, is expected to destabilize the protein. Three protein structures were utilized. First, we performed protein stability prediction based on the 3FQM structure, which has the closest reference sequence to the NS5A sequence we used in our experiments but still differs by 20 amino acid substitutions. At the residue level, we found mutations that decreased protein stability (median predicted ΔΔG change for each residue) led to reduced viral fitness (the median and mean fitness of observed mutants at each site, *P* = 7.7 × 10^−8^ and *P* = 2.3 × 10^−6^, respectively; Fig. 2D, Fig. S2C). For example, mutations at a stretch of highly conserved residues (F88 to N91) that run through the core of NS5A protein tended to destabilize the protein and significantly reduced the viral fitness. Mutations that increase ΔΔG beyond a threshold (∼5 Rosetta energy units) were mostly lethal. This is consistent with the threshold robustness model, which predicts that proteins become unfolded after using up the stability margin (15, 56, 57). The negative correlation between protein stability and viral fitness was confirmed by predicting ΔΔG using a different Protein Data Bank (PDB) model (4CL1; Fig. S2D to F). Although the sequence of 3FQM and 4CL1 has 29 amino acid differences (83.4% identity), the protein structures are highly similar to each other, and the predicted ΔΔGs are highly consistent among residues (Fig. S2D and E). Furthermore, we performed homology structural modeling using SWISS-MODEL (58) and predicted the protein structure based on the NS5A sequence we used in the experiments (Fig. S2G to I). With the same amino acid sequence, the predicted structure allowed us to compare ΔΔG and viral fitness for each individual mutant. Consistent with the result at the residue level (Fig. S2H), the negative correlation and the protein stability threshold exist for all the mutants (Fig. S2I). We also note that mutations can be deleterious because they impair protein function rather than destabilize the protein, so the correlation between protein stability and fitness is not expected to be perfect. The level of correlation between ΔΔG and fitness that we observed is similar to that from previous studies of other proteins (13, 30, 59).

10.1128/mSystems.01111-20.2 FIG S2 Mutations at amino acid sites that disrupt protein stability are highly deleterious. (A) Mutations at amino acid sites with lower solvent accessibility tend to incur larger fitness costs. The relative solvent accessibility for each residue is significantly correlated with mean relative fitness (Spearman’s ρ = 0.51, *P* = 5.1 × 10^−6^). (B) Amino acid sites that were less tolerant of mutations (average fitness of mutants, <0.2) have lower relative solvent accessibility. (C) Mutations at amino acid sites with larger effects on destabilizing protein stability (ΔΔG > 0) tend to reduce the viral replication fitness. Changes in folding free energy ΔΔG (Rosetta energy unit) of NS5A monomer were predicted using PyRosetta (PDB: 3FQM). The median ΔΔG at each amino acid site is shown. The mean fitness of observed mutants at each amino acid site is shown. (D) Alignment between two NS5A monomer structures; 4CL1 (yellow) and 3FQM (blue) are shown. The root-mean-square deviation (RMSD) between two structures is 0.631. (E) The predicted ΔΔGs based on PDB 3FQM and 4CL1 are significantly correlated (Spearman’s ρ = 0.85, *P* = 5.1 × 10^−6^). The median ΔΔG at each amino acid site is shown. (F) The negative correlation between predicted ΔΔG and replication fitness is shown with ΔΔG predicted using PDB 4CL1. The median ΔΔG and median fitness at each amino acid site are shown. (G) Protein homology modeling was performed using SWISS-MODEL with our NS5A sequence. The structural alignment between the predicted SWISS-MODEL model (magenta) and 3FQM (blue) is shown. The RMSD between two structures is 0.277. (H) The negative correlation between predicted ΔΔG and replication fitness is shown with ΔΔG predicted using SWISS-MODEL. The median ΔΔG and median fitness at each amino acid site are shown. (I) The negative correlation between predicted ΔΔG (based on SWISS-MODEL) and replication fitness is shown for each individual mutant. Download FIG S2, TIF file, 1.2 MB. Copyright © 2021 Dai et al. 2021 Dai et al. https://creativecommons.org/licenses/by/4.0/ This content is distributed under the terms of the Creative Commons Attribution 4.0 International license.

### Adaptive potential as a function of environmental stress.

Beneficial mutations are the raw materials of protein evolution (20). We aimed to study the role of environmental stress in modulating the adaptive potential of drug-targeted viral proteins. In an independent study (11), the mutant library of HCV NS5A protein was selected under a single drug concentration ([DCV] = 20 pM) to profile the effects of mutations on drug resistance. In this study, we selected the mutant library at 10, 40, and 100 pM of DCV. The drug concentrations were chosen based on the *in vitro* 50% inhibitory concentration (IC_50_) of wild-type HCV virus (∼20 pM) to represent different levels of environmental stress (mild, intermediate, and strong).

**FIG 3:**
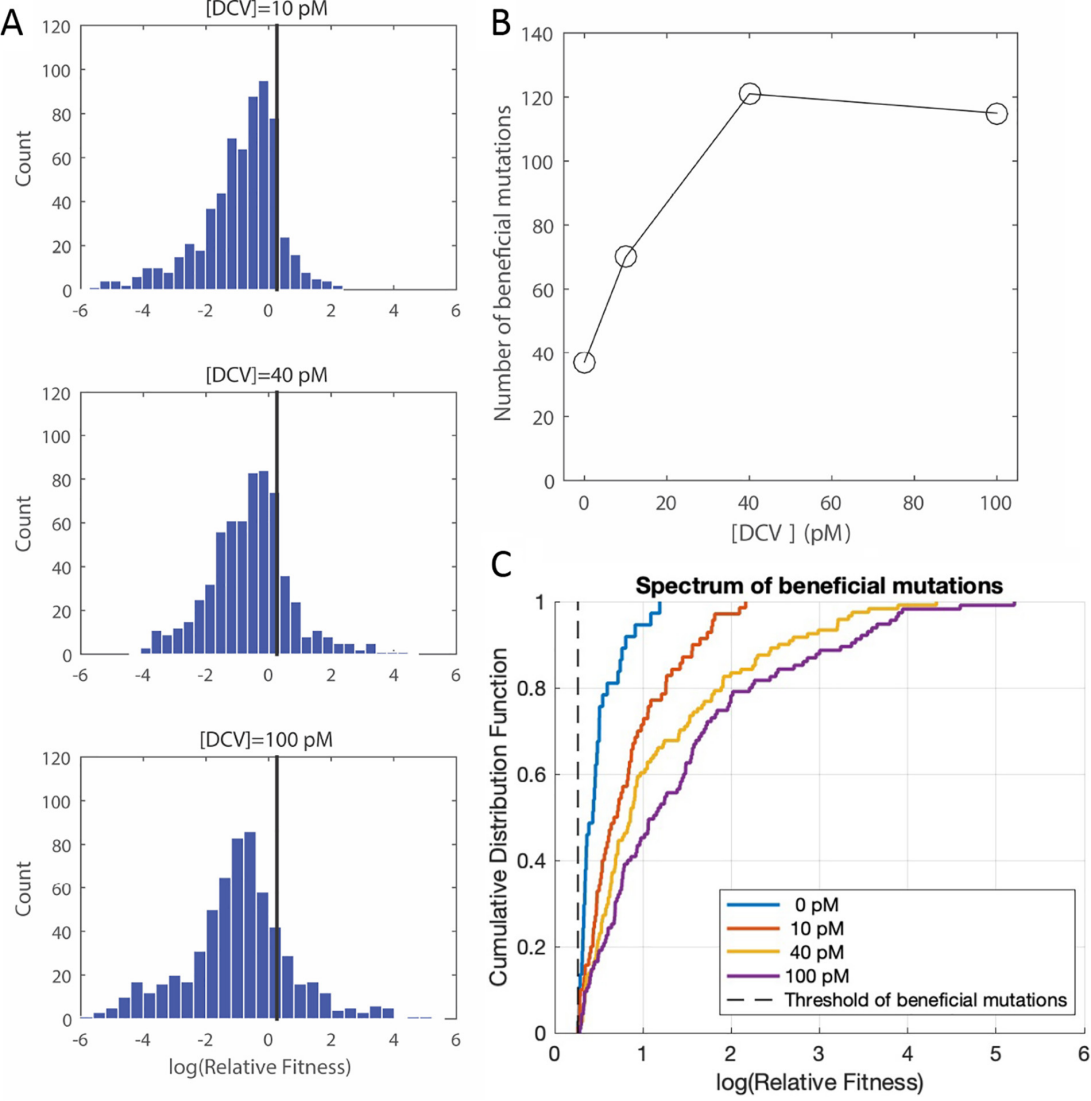
The spectrum of beneficial mutations changes under increasing environmental stress imposed by the antiviral drug daclatasvir. (A) DFE of single amino acid substitutions in domain IA of HCV NS5A protein under increasing environmental stress by daclatasvir. The black line indicates the threshold used for classifying beneficial mutations (Materials and Methods). (B) The number of beneficial mutations as a function of environmental stress imposed by daclatasvir. (C) The cumulative distribution function (CDF) of the fitness effect of beneficial mutations. The dashed black line indicates the threshold used for classifying beneficial mutations.

By tuning the concentration of DCV, we observed a change in the DFE (Table S1), particularly of beneficial mutations (Fig. 3A). At higher drug concentrations, we observed an increase in the total number of beneficial mutations (Fig. 3B, Table S2). Furthermore, the cumulative distribution function (CDF) of beneficial mutations also shows an increase in the median and maximum relative fitness (Fig. 3C). We further tested whether the shape of this distribution changed under drug selection. Previous empirical studies supported the hypothesis that the DFE of beneficial mutations is exponential or bounded on the right (40, 45, 48, 60–69). Following a maximum likelihood approach, we fit the DFE of beneficial mutations to the generalized Pareto distribution (Fig. S3; Materials and Methods). The fitted distribution is described by two parameters, a scale parameter (τ) and a shape parameter (κ) that determines the behavior of the distribution’s tail. Using a likelihood-ratio test (70), we found that our data are consistent with the null hypothesis that the DFE of beneficial mutations is exponential (κ = 0) (Table S2).

10.1128/mSystems.01111-20.3 FIG S3 The fitted distribution of fitness effects of beneficial single amino acid mutations. (A) Comparison of the observed distribution of fitness effects to the fitted distribution. Only the beneficial mutants are shown. The log transformed relative fitness for each mutant has been normalized to the beneficial threshold; thus, the curve starts from 0. (B) The exponential distribution fits the spectrum of beneficial mutations under conditions with drug selection. Download FIG S3, TIF file, 1.2 MB. Copyright © 2021 Dai et al. 2021 Dai et al. https://creativecommons.org/licenses/by/4.0/ This content is distributed under the terms of the Creative Commons Attribution 4.0 International license.

10.1128/mSystems.01111-20.7 TABLE S1 Statistics and correlations of DFE across environments. (A) The statistics are calculated using the selection coefficient of nonlethal mutations. (B) The Pearson correlation is calculated for the selection coefficient of nonlethal mutations in two different environments. Download Table S1, PDF file, 0.02 MB. Copyright © 2021 Dai et al. 2021 Dai et al. https://creativecommons.org/licenses/by/4.0/ This content is distributed under the terms of the Creative Commons Attribution 4.0 International license.

10.1128/mSystems.01111-20.8 TABLE S2 Statistics of the distribution of fitness effects of beneficial single amino acid substitutions under various selection pressures. (A) The total number of single amino acid substitutions is 1,634. In this paper, the threshold for beneficial mutations is chosen as 2σ*_silent_*, where σ*_silent_* is the standard deviation of the selection coefficients of synonymous mutations. The trend in Fig. 2 is robust to the fitness threshold for beneficial mutations. (B) The scale parameter increases at higher drug concentrations. The null hypothesis that the DFE of beneficial mutations is exponential (κ = 0) cannot be rejected (*P* > 0.05). Download Table S2, PDF file, 0.03 MB. Copyright © 2021 Dai et al. 2021 Dai et al. https://creativecommons.org/licenses/by/4.0/ This content is distributed under the terms of the Creative Commons Attribution 4.0 International license.

Furthermore, we used a maximum-likelihood approach to fit a displaced-gamma distribution to the DFE to estimate the distance to the phenotypic optimum in Fisher’s geometric model (FGM) (71, 72) (Fig. S4). The displaced-gamma distribution has the shape of a negative gamma distribution, shifted by a parameter *s*_0_ that indicates the distance of the initial genotype (i.e., wild type) to the optimum (Materials and Methods). Estimated distances to the phenotypic optimum under different conditions are summarized in Table S3. In accordance with theoretical expectations, we found that the distance to the phenotypic optimum increased as the level of environmental stress increased (i.e., increasing drug concentration).

10.1128/mSystems.01111-20.4 FIG S4 Fitted displaced-gamma distribution to the DFE. The maximum-likelihood approach was used to fit a displaced-gamma distribution to the DFE to estimate the distance to the phenotypic optimum in Fisher’s geometric model (FGM). The displaced-gamma distribution has the shape of a negative gamma distribution, shifted by a parameter *s*_0_ that indicates the distance of the initial genotype (i.e., wild type) to the optimum. The estimated shift parameters are summarized in Table S3. Download FIG S4, TIF file, 1.2 MB. Copyright © 2021 Dai et al. 2021 Dai et al. https://creativecommons.org/licenses/by/4.0/ This content is distributed under the terms of the Creative Commons Attribution 4.0 International license.

10.1128/mSystems.01111-20.9 TABLE S3 Estimated distances (95% confidence interval [CI]) to the optimum under the assumption of a displaced-gamma distribution. The shift parameter *s*_0_ indicates the distance of the initial genotype (i.e., wild-type) to the optimum in Fisher’s geometrical model. Download Table S3, PDF file, 0.03 MB. Copyright © 2021 Dai et al. 2021 Dai et al. https://creativecommons.org/licenses/by/4.0/ This content is distributed under the terms of the Creative Commons Attribution 4.0 International license.

### A pharmacodynamics model explains the shift of DFE with increased drug concentration.

**FIG 4:**
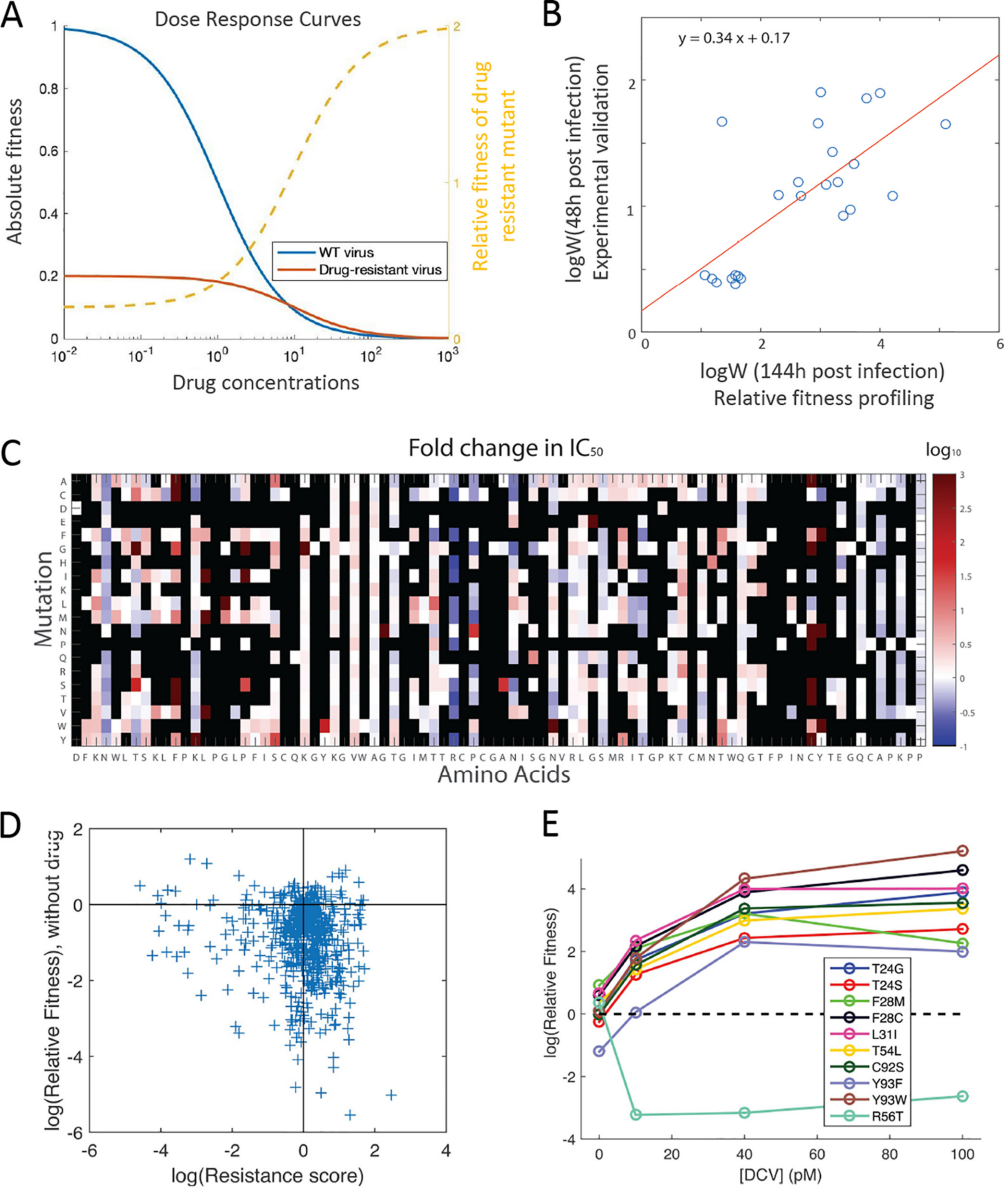
The adaptive potential under drug selection is determined by the effects of mutations on replication fitness and drug resistance. (A) Hypothetical dose response curves of the wild-type (WT) virus and a drug-resistant mutant virus. The blue and red lines represent the absolute fitness of WT virus and drug-resistant mutant virus, respectively. The yellow dashed line represents the relative fitness of the mutant virus to WT virus. The absolute fitness decreases with drug concentration [*drug*] following $f=f_{0}\frac{IC_{50}}{IC_{50}+[\mathrm{drug}]}$, where *f*_0_ is the fitness without drug selection and IC_50_ is the half inhibitory concentration. Compared to the wild-type virus, the hypothetical drug-resistant mutant carries a fitness cost (smaller *f*_0_) but is less sensitive to drug inhibition (larger IC_50_). The relative fitness of the drug-resistant mutant is expected to increase with drug concentration. When drug concentration → ∞, the *RF_mut_*_[_*_drug_*_]_ → *f*_0_ *_Mut_IC*_50_(*mut*)*IC*_50(_*_mut_*_)_/*f*_0_ *_WT_ IC*_50(_*_WT_*_)_. In the hypothetical curve, we set *f*_0_ *_WT_* = 1, *IC*_50(_*_WT_*_)_ = 1; *f*_0_ *_Mut_* = 0.2, *IC*_50(_*_Mut_*_)_ = 10. Then *RF_mut_*_[_*_drug_*_]_ would approach 0.2 · 10 = 2 when drug concentration → ∞. The hypothetical curves explain the increase of beneficial mutations upon drug treatment. (B) The drug resistance score *W* estimated from validation experiments of individual mutants is consistent with the estimates based on the pharmacodynamics modeling of the screening result (Pearson correlation = 0.71, *P* = 1.1 × 10^−4^). As the experiment collected virus at 48 h postinfection while the screening cultured for 144 h, the ratio between log(W_experimental validation_) and log(W_fitness profiling_) is expected to be 48 h/144 h = 0.33 under exponential growth. The fitted linear curve (red line) gives a ratio of 0.34, which is consistent with the expectation. (C) The heatmap shows the predicted IC_50_ value of all mutants. Lethal mutations are marked with black. (D) The effects of mutations on replication fitness (i.e., fitness without drug) and drug resistance score *W* at [DCV] = 40 pM are shown by the scatterplot. (E) Relative fitness of the validated drug-resistant and drug-sensitive mutants (Fig. S5) as a function of [DCV]. With the increase of drug concentration, the relative fitness of the drug-resistant mutant is increased.

Our results show that the adaptive potential of proteins is modulated by the strength of environmental stress. To explain the changing spectra of beneficial mutations upon drug treatment, we employed a pharmacodynamics model describing viral fitness as a function of drug concentration (i.e., phenotype-fitness mapping) (Fig. 4A).

$$f=f_{0}\frac{IC_{50}}{IC_{50}+[\mathrm{drug}]}$$

10.1128/mSystems.01111-20.5 FIG S5 Dose response curve of validated mutants (10 drug-resistant mutants, 1 drug-sensitive mutant) and WT virus. The Hill coefficient describing the sigmoidal shape of the dose response curve is fixed to 1, as used in fitting the dose response curves of wild-type virus and validated mutant viruses. The unit of IC_50_ is pM. The virus titer was measured after 48 h of growth under drug treatment (see Methods in reference 11). Download FIG S5, TIF file, 1.2 MB. Copyright © 2021 Dai et al. 2021 Dai et al. https://creativecommons.org/licenses/by/4.0/ This content is distributed under the terms of the Creative Commons Attribution 4.0 International license.

where *f*_0_ is the fitness without drug selection and IC_50_ is the half inhibitory concentration. The absolute fitness *f* decreases with drug concentration ([drug]). In this model, the fitness of each mutant under drug selection is contributed by two traits, the fitness without drug selection (*f*_0_) and the effect on drug resistance (IC_50_). We define the drug resistance score (W) of a mutant as the ratio of the relative fitness under drug selection to that without drug selection.

Wmut,[drug]=(fmutfwt)/f0 mutf0 wt

Based on the above pharmacodynamics model, *W* is proportional to the IC_50_ of the mutant. To examine the accuracy of *W* using an experimentally validated dose response curve, we utilized a set of mutants previously constructed by site-directed mutagenesis (Fig. S5) (11). The drug dose response curves were experimentally measured for each individual mutant. We found that the effects of mutations on drug resistance (*W*) estimated from the fitness data were generally consistent with estimates based on the measured dose response curves (Fig. 4B and Fig. S6; Materials and Methods), suggesting that the drug resistance score *W* is accurate and can be used to estimate IC_50_. Thus, we estimated the IC_50_ value of all profiled mutations (Fig. 4C). We found that residues 28, 31, 92, and 93 are enriched with drug resistance mutations with high IC_50_ values, consistent with a previous experimental study (11). These positions were also reported to be hot spots for DCV drug resistance in multiple HCV genotypes (73–75).

10.1128/mSystems.01111-20.6 FIG S6 Drug resistance can be inferred from fitness data under drug selection. The scatter plot shows that the drug resistance (W) estimated from different selection conditions (different concentrations of DVC) is highly correlated. Download FIG S6, TIF file, 1.2 MB. Copyright © 2021 Dai et al. 2021 Dai et al. https://creativecommons.org/licenses/by/4.0/ This content is distributed under the terms of the Creative Commons Attribution 4.0 International license.

This pharmacodynamics model can help explain the change of DFE with the increase of drug concentration. The mutations that reduce a protein’s binding affinity to drug molecules (i.e., less inhibited by the drug) may come with a fitness cost (i.e., smaller *f*_0_ than the wild type). Among all the nonlethal single amino acid substitutions profiled in our HCV NS5A protein library, we found that roughly half of the mutations increased resistance to DCV (i.e., improved new function) at the expense of replication fitness without drug (Fig. 4D; Spearman’s ρ = –0.13, *P* = 8.3 × 10^−4^). This group of resistance mutations (lower-right section in Fig. 4D) can become beneficial when the environmental stress imposed by the antiviral drug is strong, leading to an increase in the proportion of beneficial mutations at higher drug concentrations. Moreover, as the wild-type virus moves further away from the phenotypic optimum, the relative fitness of the drug-resistant mutant is expected to increase with environmental stress (Fig. 4A, dashed line). Indeed, we found that the relative fitness of validated drug-resistant mutants increased at higher drug concentration (Fig. 4E).

## DISCUSSION

Site-directed mutagenesis and experimental evolution are traditional approaches to examine the DFE (76–79). Both methods provide pivotal insights into the shape of the DFE, yet with limitations. The site-directed mutagenesis approach requires fitness assays for each individual mutant and can only provide a sparse sampling of mutations. In experimental evolution, the sampling of sequence space via *de novo* mutations is biased toward large-effect beneficial mutations, as they are more likely to fix in the population. In contrast, the deep mutational scanning approach (9), which utilizes high-throughput sequencing to simultaneously assay the fitness or phenotype of a library of mutants, allows for unbiased and large-scale sampling of fitness landscapes and thus is ideal for studying the characteristics of empirical DFE. The downside of this high-throughput approach is that the fitness measurements can be noisy, especially for large mutant libraries (80). In our experiment, we divided the mutant library into smaller sublibraries (∼500 mutants) in selection experiments. We compared the data to an independent experiment and found that the fitness estimates were largely reproducible (Fig. S2). We also showed that the observed change in the DFE under different conditions was consistent with validation experiments (Fig. 3). Since this study is focused on the properties of the entire distribution of mutations rather than the effects of specific mutations, our findings on the general patterns of DFE are robust to the errors in fitness estimates. Our study quantified the fitness effects of single amino acid substitutions in the drug-targeted region of an essential viral protein. In general, the empirical DFE of HCV NS5A was consistent with previous findings that viral proteins were highly optimized in the natural condition and very sensitive to the effects of deleterious mutations.

One crucial point is that DFE will vary as a function of the environment (33, 35, 81). In the study by Stiffler, the level of environmental stress is controlled by ampicillin concentration (33). Because TEM-1’s function is to degrade ampicillin, deleterious mutations that impair the enzyme function (“loss-of-function”) would become more deleterious at higher dose of ampicillin. In our system, we expect that the function of HCV NS5A protein for viral replication and drug resistance to daclatasvir are two relatively independent traits; thus, the dose of daclatasvir should not alter the strength of purifying selection on maintaining protein stability and viral replication. Indeed, we do not find much difference on the deleterious side of DFE across different environments. Instead, we have observed significant changes on the beneficial side of DFE as a function of the drug dose. Because HCV NS5A protein is not well adapted in the novel environment of daclatasvir selection, the effect of drug resistance mutations (“gain-of-function”) becomes more beneficial at higher drug dose. Moreover, due to the pleiotropic effect of mutations on drug resistance and replication fitness (Fig. 4), there is an increasing supply of beneficial mutations at higher drug dose.

Although different systems have distinct protein-drug interactions that lead to different resistance profiles (82), the results in our study provide a general framework to study the DFE of drug-targeted proteins. Future studies along this line will further our understanding of how proteins evolve new functions under the constraint of maintaining their original function (83), as exemplified in the evolution of resistance to directly acting antiviral drugs (84). Quantifying the characteristics of the DFE of drug-targeted proteins in different environments (e.g., varying levels of environmental stress or conflicting selection pressures) would allow us to assess repeatability in the outcomes of viral evolution (85) and guide the design of therapies to minimize drug resistance (34).

## MATERIALS AND METHODS

### Mutagenesis.

The mutant library of HCV NS5A protein domain IA (86 amino acids) was constructed using saturation mutagenesis as previously described (11). In brief, the entire region was divided into five sublibraries, each containing 17 to 18 amino acids (∼500 mutants in each sublibrary). NNK (N: A/T/C/G, K: T/G) was used to replace each amino acid. The oligos, each of which contains one random codon, were synthesized by Integrated DNA Technologies (IDT). The mutated region was ligated to the flanking constant regions, subcloned into the pFNX-HCV plasmid, and then transformed into bacteria. The pFNX-HCV plasmid carrying the viral genome was synthesized in Ren Sun’s lab based on the chimeric sequence of genotype 2a HCV strains J6/JFH1.

### Cell culture.

The human hepatoma cell line (Huh-7.5.1) was provided by Francis Chisari from the Scripps Research Institute, La Jolla, California. The cells were cultured in T-75 tissue culture flasks (Genesee Scientific) at 37°C with 5% CO_2_. The complete growth medium contained Dulbecco’s modified Eagle’s medium (Corning Cellgro), 10% heat-inactivated fetal bovine serum (Omega Scientific), 10 mM HEPES (Life Technologies), 1× minimal essential medium (MEM) nonessential amino acids solution (Life Technologies) and 1× penicillin-streptomycin-glutamine (Life Technologies).

### Selection of mutant viruses.

The plasmid mutant library was transcribed *in vitro* using a T7 RiboMAX Express large scale RNA production system (Promega) and purified using a PureLink RNA minikit (Life Technologies). Then, 10 μg of *in vitro* transcribed RNA was used to transfect 4 million Huh-7.5.1 cells via electroporation using Bio-Rad Gene Pulser (246 V, 950 μF). The supernatant was collected 6 days posttransfection, and virus titer was determined by immunofluorescence assay. The viruses collected after transfection were used to infect ∼2 million Huh-7.5.1 cells with an multiplicity of infection (MOI) of around 0.1 to 0.2. The five sublibraries were passaged for selection separately. For the three different levels of selection pressure, the growth medium was supplemented with 10 pM, 40 pM, and 100 pM HCV NS5A inhibitor daclatasvir (BMS-790052), respectively. The supernatant was collected at 6 days postinfection.

### Preparation of Illumina sequencing samples.

For each sample, viral RNA was extracted from 700 μl supernatant collected after transfection and after selection using a QIAamp viral RNA minikit (Qiagen). Extracted RNA was reverse transcribed into cDNA with a SuperScript III reverse transcriptase kit (Life Technologies). The targeted region in NS5A (51 to 54 nucleotides [nt]) was PCR amplified using KOD Hot Start DNA polymerase (Novagen). The Eppendorf thermocycler was set as follows: 2 min at 95°C; 25 to 35 three-step cycles of 20 s at 95°C, 15 s at 52 to 56°C (sublibrary 1, 52°C; 2, 52°C; 3, 52°C; 4, 56°C; 5, 54°C), and 25s at 68°C; 1 min at 68°C. The number of PCR cycles was chosen based on the copy number of cDNA templates as determined by quantitative PCR (qPCR) (Bio-Rad). The PCR products were purified using a PureLink PCR purification kit (Life Technologies) and prepared for Illumina HiSeq 2000 sequencing (paired-end, 100 bp) following 5′-phosphorylation using T4 polynucleotide kinase (New England BioLabs), 3′ dA-tailing using a dA-tailing module (New England BioLabs), and TA ligation of the adapter using T4 DNA ligase (Life Technologies). Each sample was tagged with unique 3-bp customized barcodes, which were part of the adapter sequence and were sequenced as the first three nucleotides in both the forward and reverse reads (59).

### Analysis of Illumina sequencing data.

The sequencing data were parsed using the SeqIO function of BioPython. The reads from different samples were demultiplexed by the barcodes and mapped to the entire mutated region in NS5A by allowing, at maximum, 5 mismatches with the reference genome (11). Since both forward and reverse reads cover the whole amplicon, we used paired reads to correct for sequencing errors. A mutation was called only if it was observed in both reads and the quality score at the corresponding position was at least 30. Sequencing reads containing mutations not supposed to appear in our single-codon mutant library were excluded from downstream analysis. The sequencing depth for each sublibrary is at least ∼10^5^ and 2 orders of magnitude higher than the library complexity.

### Calculation of relative fitness.

For each condition of selection experiments (i.e., different concentration of daclatasvir [DCV]), the relative fitness (RF) of a mutant virus to the wild-type virus was calculated by the relative changes in frequency after selection,

$$RF_{\mathrm{mut}}([\mathrm{DCV}])=\left(\frac{f_{\mathrm{mut}}^{T=2}}{f_{\mathrm{mut}}^{T=1}}\right)/\left(\frac{f_{\mathrm{WT}}^{T=2}}{f_{\mathrm{WT}}^{T=1}}\right)$$

where $f_{\mathrm{mut}}^{T=\mathrm{round}}$ and $f_{\mathrm{WT}}^{T=\mathrm{round}}$ is the frequency of the mutant virus and the wild-type virus at round 1 (after transfection) or round 2 (after infection). The fitness of wild-type virus is normalized to 1. The fitness values estimated from one round (round 1 to round 2) have been shown to be highly consistent with estimates based on round 0 to round 1 (Fig. S2) and estimates from multiple rounds of selection (11). A mutant was labeled as “missing” if the mutant’s frequency in the plasmid library was less than 0.0005. A mutant was labeled as “lethal” if the mutant’s frequency after transfection was less than 0.0005 or its frequency after infection was 0 (RF = 0) (11).

The threshold for beneficial mutations was chosen as 2σ*_silent_*, where 2σ*_silent_* is the standard deviation of the log transformed RF of synonymous mutations (Fig. 1). The fitness effects of nonsynonymous mutations leading to the same amino acid substitution were averaged to estimate the fitness effect of the given single amino acid substitution.

### Fitting the distribution of fitness effects of beneficial mutations.

The distribution of log transformed RF of beneficial mutations was fitted to a generalized Pareto distribution following a maximum likelihood approach (70):

F(x|κ,τ)={1−(1+κτx)−1κ,x≥0, if κ > 0(Frechet)1−(1+κτx)−1κ,0≤x<−τκ, if κ < 0 (Weibull)1−e−xτ,x≥0, if κ = 0 (Gumbel)

Only mutations with RF higher than the beneficial threshold 2σ*_silent_* were included in the distribution of beneficial mutations. The RFs were normalized to the beneficial threshold. The shape parameter κ determines the tail behavior of the distribution, which can be divided into three domains of attraction, Gumbel domain (exponential tail, κ = 0), Weibull domain (truncated tail, κ < 0), and Fréchet domain (heavy tail, κ > 0). For each selection condition, a likelihood ratio test was performed to evaluate whether the null hypothesis κ = 0 (exponential distribution) can be rejected.

### Fitting the distribution of fitness effects to Fisher’s geometrical model.

Fisher’s geometrical model predicts that the distribution of fitness effects of mutations is distributed according to a negative displaced gamma distribution (71, 72). This distribution has a shape parameter (α), a scale parameter (β), and a displacement parameter (*s_0_*). We assume that RFs are measured with a normally distributed measurement error with standard deviation σ*_silent_*. Thus, the observed distribution of RFs is modeled as the sum of a gamma and normally distributed random variable. We used the NormalGamma package in R to numerically compute the normal-gamma density function (86). Maximum likelihood estimates of the parameters of the negative displaced gamma distribution were obtained with L-BFGS-B optimization implemented in the R function optim.

### Inferring drug resistance from fitness data.

We can quantify the drug resistance of each mutant in the library by computing its fold change in relative fitness,

$$W([\mathrm{DCV}])=\frac{RF_{\text{mut}}([\mathrm{DCV}])}{RF_{\text{mut}}}$$

Here, *RF*_mut_ is the relative fitness of a mutant under the natural condition (i.e., no drug). *W* is the fold change in relative fitness and represents the level of drug resistance relative to the wild type. *W* > 1 indicates drug resistance, and *W* < 1 indicates drug sensitivity.

This empirical measure of drug resistance can be directly linked to a simple pharmacodynamics model (84), where the viral replicative fitness is modeled as a function of drug dose,

$$W_{\text{predict}}([\mathrm{DCV}])=\left(\frac{IC_{\text{mut}}}{[\mathrm{DCV}]+IC_{\text{mut}}}\right)/\left(\frac{IC_{\text{wt}}}{[\mathrm{DCV}]+IC_{\text{wt}}}\right)$$

Here, *IC* denotes the half-inhibitory concentration. The Hill coefficient describing the sigmoidal shape of the dose response curve is fixed to 1, as used in fitting the dose response curves of wild-type virus and validated mutant viruses. The drug resistance score *W* inferred from fitness data is consistent with the drug resistance score *W*_predict_ predicted from dose response curves of validated mutants (Fig. S6).

### Calculation of relative solvent accessibility.

DSSP (https://swift.cmbi.umcn.nl/gv/dssp/DSSP_3.html) was used to compute the solvent accessible surface area (SASA) (87) from the HCV NS5A protein structure (PDB: 3FQM) (52). The SASA was then normalized to relative solvent accessibility (RSA) using the empirical scale reported in reference 88.

### Predictions of protein stability.

Homology modeling based on our NS5A sequence was performed using the SWISS-MODEL server (58) (https://swissmodel.expasy.org/).

The ΔΔG (in Rosetta energy units) of HCV NS5A mutants was predicted using PyRosetta (version PyRosetta4.conda.mac.python37.Release r242) as the difference in scores between the monomer structure of mutants (single amino acid mutations from sites 32 to 103) and the reference (PDB: 3FQM; 4CL1 or the homology model). The score is designed to capture the change in thermodynamic stability caused by the mutation (ΔΔG).

The PDB file of the NS5A dimer was cleaned and trimmed to a monomer (chain A). Next, all side chains were repacked (sampling from the 2010 Dunbrack rotamer library [88]) and minimized for the reference structure using the “ddg_monomer” scoring function. After an amino acid mutation was introduced, the mutated residue was repacked, followed by line minimization of the backbone and all side chains (algorithm, “linmin”). This procedure was performed 10 times, and the predicted ΔΔG of a mutant structure is the average of all the scoring structures.

We note that predictions based on the NS5A monomer structure were only meant to provide a crude profile of how mutations at each site may impact protein stability. Potential structural constraints at the dimer interface have been ignored, which is further complicated by the observations of two different NS5A dimer structures (51, 52).

### Diversity of HCV sequences identified in patients.

Aligned nucleotide sequences of HCV NS5A protein were downloaded from the Los Alamos National Lab database (89) (all HCV genotypes, ∼2,600 sequences total) and clipped to the region of interest (amino acids 18 to 103 of NS5A). Sequences that caused gaps in the alignment of the H77 reference genome were manually removed. After translation to amino acid sequences, sequences with ambiguous amino acids were removed (∼2,300 amino acid sequences after filtering). The sequence diversity at each amino acid site was quantified by Shannon entropy. The frequency of amino acid on each site that differs from our NS5A sequence was calculated.

### Data and reagent availability.

All research materials are available upon request. Raw sequencing data have been submitted to the NIH Sequence Read Archive (SRA) under BioProject number PRJNA395730. All scripts have been deposited at https://github.com/leidai-evolution/DFE-HCV.

### Ethics statement.

The use of human cell lines and infectious agents in this paper is approved by the Institutional Biosafety Committee at the University of California, Los Angeles (IBC no. 40.10.2-f).

## Supplementary Material

Reviewer comments
