# Supplementary figures and images for: Quantifying the Evolutionary Constraints and Potential of Hepatitis C Virus NS5A Protein
Source: mSystems. 2021 Apr 13;6(2):e01111-20. doi: 10.1128/mSystems.01111-20 (PMC8546995; doi:10.1128/mSystems.01111-20)

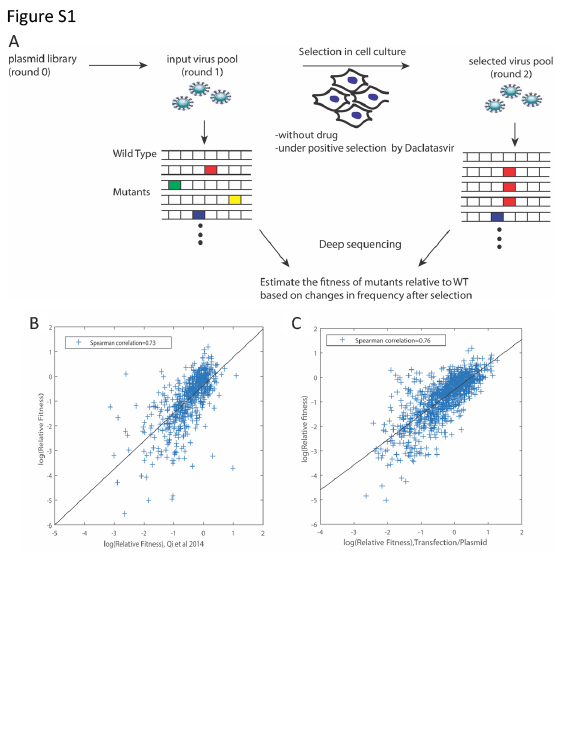

Supplement: FIG S1 [file msystems.01111-20-sf001.tif]

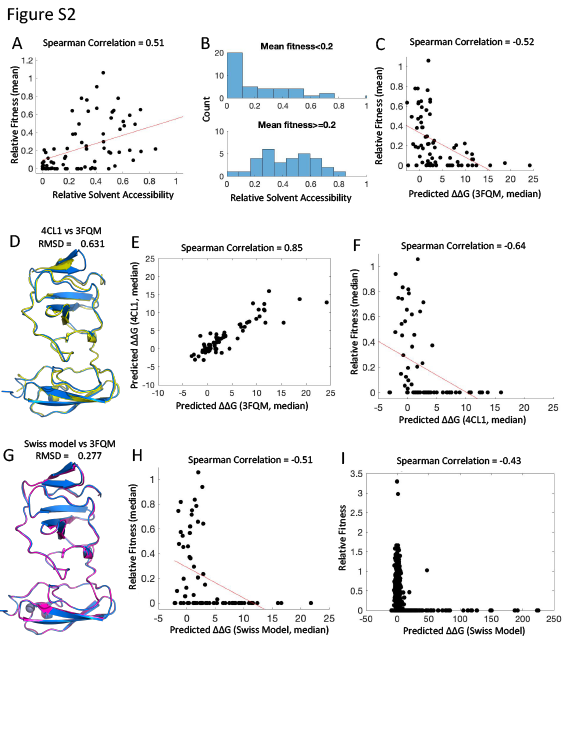

Supplement: FIG S2 [file msystems.01111-20-sf002.tif]

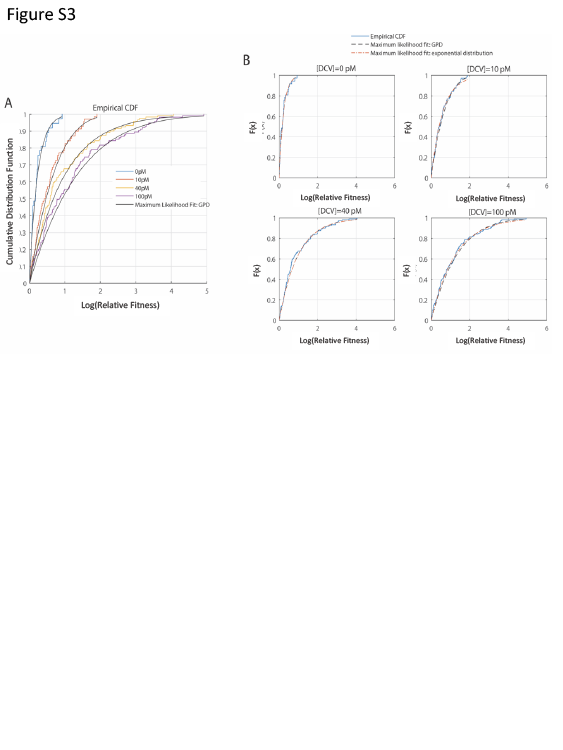

Supplement: FIG S3 [file msystems.01111-20-sf003.tif]

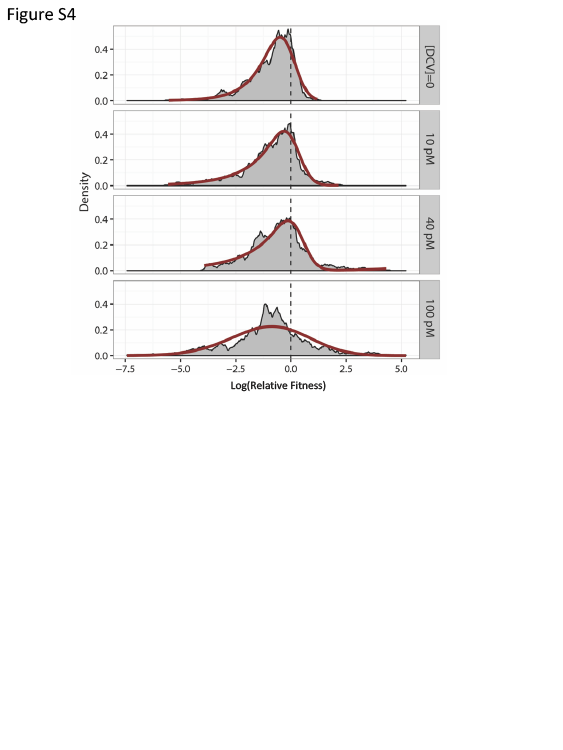

Supplement: FIG S4 [file msystems.01111-20-sf004.tif]

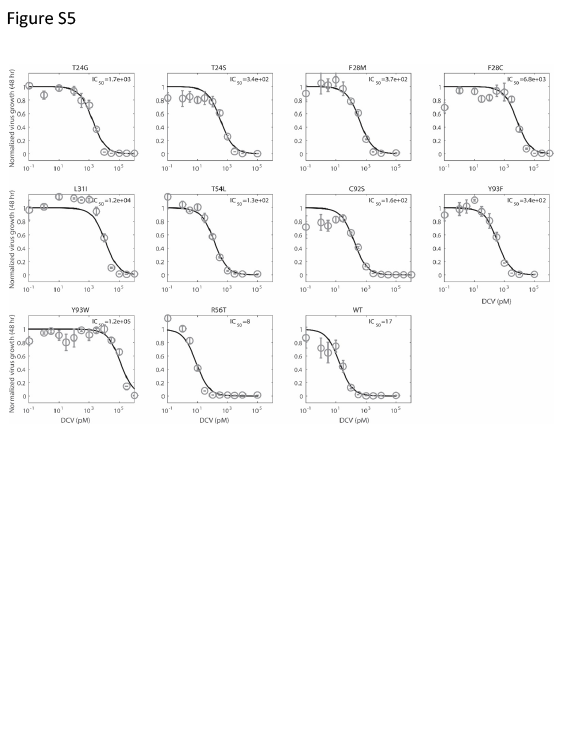

Supplement: FIG S5 [file msystems.01111-20-sf005.tif]

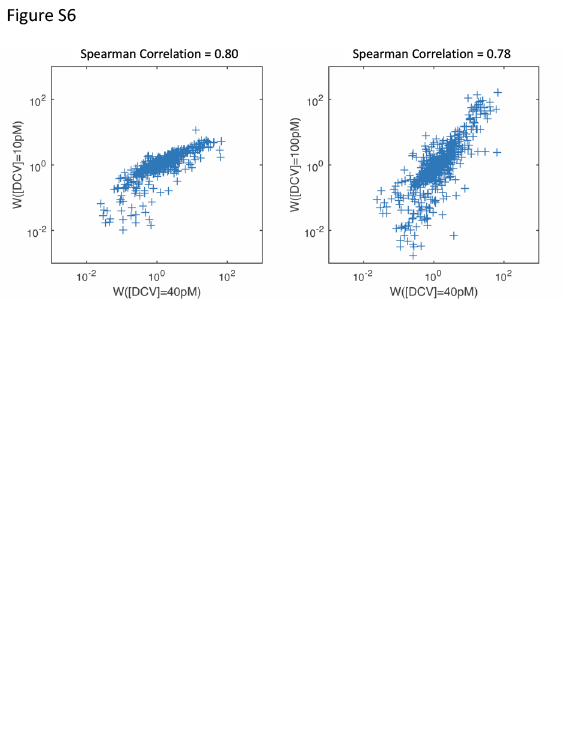

Supplement: FIG S6 [file msystems.01111-20-sf006.tif]
