# Supplementary material for: Quantifying the Evolutionary Constraints and Potential of Hepatitis C Virus NS5A Protein
Source: mSystems. 2021 Apr 13;6(2):e01111-20. doi: 10.1128/mSystems.01111-20 (PMC8546995; doi:10.1128/mSystems.01111-20)
Supplement: TABLE S1 [file msystems.01111-20-st001.pdf]

Tables S1

(A)

[DCV]	Mean	Variance	Skewness	Kurtosis
0 pM	-0.89	0.99	-1.19	4.83
10 pM	-0.92	1.61	-0.94	4.23
40 pM	-0.60	1.64	0.25	4.05
100 pM	-1.01	3.12	-0.13	4.17

(B)

[DCV]	0 pM	10 pM	40 pM	100 pM
0 pM	1	0.69	0.43	0.28
10 pM		1	0.80	0.70
40 pM			1	0.80
100 pM				1
