# Supplementary material for: Quantifying the Evolutionary Constraints and Potential of Hepatitis C Virus NS5A Protein
Source: mSystems. 2021 Apr 13;6(2):e01111-20. doi: 10.1128/mSystems.01111-20 (PMC8546995; doi:10.1128/mSystems.01111-20)
Supplement: TABLE S2 [file msystems.01111-20-st002.pdf]

Table S2

(A)

**[DCV]**	s>2σ_{silent}	s>σ_{silent}	s>0
0 pM	37	63	97
10 pM	70	96	141
40 pM	121	147	182
100 pM	115	127	141

(B)

**[DCV]**	**Number of beneficial single amino acid substitutions**	**Scale parameter τ**	**Shape parameter κ**	**p-value**
0 pM	37	0.22	-0.01	0.95
10 pM	70	0.73	-0.23	0.16
40 pM	121	0.97	0.00	0.99
100 pM	115	1.39	-0.12	0.36
