# Supplementary material for: Quantifying the Evolutionary Constraints and Potential of Hepatitis C Virus NS5A Protein
Source: mSystems. 2021 Apr 13;6(2):e01111-20. doi: 10.1128/mSystems.01111-20 (PMC8546995; doi:10.1128/mSystems.01111-20)
Supplement: TABLE S3 [file msystems.01111-20-st003.pdf]

Table S3

[DCV]	Shift parameter s_0
0 pM	0 [-0.22, 0.22]
10 pM	0.17 [-0.11, 0.46]
40 pM	0.38 [-0.25, 1.00]
100 pM	17.22 [13.43, 21.01]
